# Detection and Localization of Myocardial Infarction Based on Multi-Scale ResNet and Attention Mechanism

**DOI:** 10.3389/fphys.2022.783184

**Published:** 2022-01-28

**Authors:** Yang Cao, Wenyan Liu, Shuang Zhang, Lisheng Xu, Baofeng Zhu, Huiying Cui, Ning Geng, Hongguang Han, Stephen E. Greenwald

**Affiliations:** ^1^School of Intelligent Medicine, China Medical University, Shenyang, China; ^2^College of Medicine and Biological Information Engineering, Northeastern University, Shenyang, China; ^3^Key Laboratory of Medical Image Computing, Ministry of Education, Shenyang, China; ^4^Neusoft Research of Intelligent Healthcare Technology, Co., Ltd., Shenyang, China; ^5^Department of Cardiology, Shengjing Hospital of China Medical University, Shenyang, China; ^6^Department of Cardiac Surgery, General Hospital of Northern Theater Command, Shenyang, China; ^7^Barts and the London School of Medicine and Dentistry, Blizard Institute, Queen Mary University of London, London, United Kingdom

**Keywords:** myocardial infarction, multi-lead ECG, residual network, attention mechanism, gradient class activation mapping

## Abstract

**Purpose:**

Myocardial infarction (MI) is one of the most common cardiovascular diseases, frequently resulting in death. Early and accurate diagnosis is therefore important, and the electrocardiogram (ECG) is a simple and effective method for achieving this. However, it requires assessment by a specialist; so many recent works have focused on the automatic assessment of ECG signals.

**Methods:**

For the detection and localization of MI, deep learning models have been proposed, but the diagnostic accuracy of this approaches still need to be improved. Moreover, with deep learning methods the way in which a given result was achieved lacks interpretability. In this study, ECG data was obtained from the PhysioBank open access database, and was analyzed as follows. Firstly, the 12-lead ECG signal was preprocessed to identify each beat and obtain each heart interval. Secondly, a multi-scale deep learning model combined with a residual network and attention mechanism was proposed, where the input was the 12-lead ECG recording. Through the SENet model and the Grad-CAM algorithm, the weighting of each lead was calculated and visualized. Using existing knowledge of the way in which different types of MI gave characteristic patterns in specific ECG leads, the model was used to provisionally diagnose the type of MI according to the characteristics of each of the 12 ECG leads.

**Results:**

Ten types of MI anterior, anterior lateral, anterior septal, inferior, inferior lateral, inferior posterior, inferior posterior lateral, lateral, posterior, and posterior lateral were diagnosed. The average accuracy, sensitivity, and specificity for MI detection of all lesion types was 99.98, 99.94, and 99.98%, respectively; and the average accuracy, sensitivity, and specificity for MI localization was 99.79, 99.88, and 99.98%, respectively.

**Conclusion:**

When compared to existing models based on traditional machine learning methods, convolutional neural networks and recurrent neural networks, the results showed that the proposed model had better diagnostic performance, being superior in accuracy, sensitivity, and specificity.

## Introduction

Myocardial infarction (MI) resulting from coronary artery occlusion causes rapid and irreversible myocardial injury and before the event, the occlusive lesion often causes no warning symptoms. According to the World Health Organization, coronary heart disease is the main cause of MI, accounting for approximately one third of deaths in those over 35 years old (Roger, 2007). It is estimated that an additional 155,000 silent first MIs, i.e., those with few, if any symptoms, occur every year (Benjamin et al., 2018).

The early detection of MI is of great value in averting complications such as heart failure, and arrhythmia, as well as death. The ECG is a rich source of information about the performance of the heart and is the most widely used means to diagnose cardiovascular disease (Sharma and Sunkaria, 2018, 2020; Zhang and Li, 2019). In patients, at risk of cardiovascular disease, the ECG signal may depart from its normal pattern in a way which can reveal the presence of abnormal heart activity, often before overt symptoms are seen. Thus, the ECG is important for the early diagnosis of a variety of cardiovascular pathologies, including MI. However, to analyze the ECG signal is time consuming and requires specialist knowledge. In recent years, machine learning and deep learning have become widely used for disease diagnosis, including cardiovascular disorders such as arrhythmia (Wang et al., 2010; Oh et al., 2019; Yao et al., 2020). For the diagnosis and prediction of MI, many machine learning models have been proposed. Dohare et al. (2018) extracted 220 features of the ECG signal including P wave duration, QRS duration, ST-T composite interval and QT interval, and obtained 14 features by principal component analysis, which were then input into a support vector machine (SVM) model to detect MI. Diker et al. (2018) used 23 features extracted from the morphology, statistics and discrete wavelet transform of ECG signals, and employed a genetic algorithm to reveal the most relevant features. In the process, the dimensionality of the feature set was reduced from 23 to 9. Finally, a SVM model was employed to classify the features. Sharma et al. (2018) decomposed the ECG signal into six sub-bands by the wavelet transform, and then extracted three features such as entropy, signal fractal dimension and Rennie entropy from the sub-bands. Following this the features were input into the k-nearest neighbors algorithm (KNN) for the diagnosis of MI. Acharya et al. (2016) extracted four kinds of features from different decomposition bands using leads II, III, and AVF, these being sample entropy, normalized sub-band energy, energy entropy, and average slope. They were input into the SVM, KNN, and the inert algorithm and their performance was compared. Sharma et al. (2018) extracted three time-domain features of each beat (T-wave amplitude, Q-wave amplitude, and standard deviation) and combined them with the 12-lead data to form a 36-dimensional feature vector, which was input to a KNN classifier again for MI diagnosis. Bhaskar (2015) proposed an artificial neural network based on preprocessing, the wavelet transform and principal component analysis to obtain the features of ECG signals for classification. The performance was analyzed by a back propagation neural network and a support vector machine. Machine learning has achieved promising results, but there is still much room for improvement.

In recent years, deep learning has been widely used in the diagnosis and prediction of MI. Convolutional neural networks (CNN) have achieved notable success. Acharya et al. (2017) and Baloglu et al. (2019) proposed a 10-layer and 11-layer CNN to detect MI. Reasat and Shahnaz (2017) took three-lead ECG signal segments and the signal from each lead was input to an induction module. Tripathy et al. (2019a) have used 12-lead ECG data for the detection of MI from Physikalisch-Technische Bundesanstalt (PTB) diagnostic ECG database based on the deep layer least-square SVM. The CNN was used for used for the localization of MI. The extracted features were then fed into a global average pooling layer and finally classified by a SoftMax layer (Tripathy et al., 2019b). In addition, recurrent neural networks (RNN) have achieved success in processing time series signals. Long short-term memory (LSTM), with its inherent suitability in processing time series signals, provides higher diagnostic accuracy in combination with a CNN than by using CNN alone. Lui and Chow (2018) proposed a CNN-LSTM model to detect MI. Because of its gate structure, LSTM effectively solves the long-term dependence problem in standard recurrent neural networks. Although the aforementioned methods have met with some success, they rely on extracting features manually, which leads to poor generalization performance and insufficient accuracy for clinical requirements. Moreover, deep learning methods obscure the link between the ECG findings and the underlying pathophysiology.

This study was motivated by the limited performance of current methods for diagnosing MI from the characteristics of ECG signals as well as the lack of feature analysis obtained by deep learning methods. To overcome these limitations, an ECGNet model which contains a multi-scale ResNet based on residual blocks, combined with the attention mechanism approach is proposed for detecting and localizing MIs. Specifically, the two main motivations were: firstly, to extract the characteristic information each dimension using a multi-scale model and to analyze the effect on the various ECG leads of different types of myocardial infarction. The second was to further improve the accuracy of the model. When compared to other approaches based on deep learning, the proposed method provided more abundant features from the ECG signals. The main innovations are as follows:

(1) One-dimensional ECG data is treated as a two-dimensional, and the same convolution kernel is used in processing the signals from different leads, thus making full use of the similarity between the different leads, and reducing the number of parameters without sacrificing accuracy.
(2) For the same lead, different sizes of convolution kernel are used to extract diverse features and fuse them to achieve intelligent detection and accurate positioning of the MI.
(3) Focusing on the influence of different leads on the localization of MI, the SENet model is added to automatically calculate the weighting assigned to each lead for each type of MI immediately before the data is input into the network and after convolution of different scales. Thus, the effect of different leads can be analyzed by visualizing these weights.
(4) The gradient class activation mapping (Grad-CAM) algorithm is used to calculate the contribution of each lead to the localization of different types of MI and is combined with an explanation based on clinical experience and knowledge of MI pathology to provide an accurate diagnosis.

The article is organized as follows: Section 2 describes the materials and methods. Section 3 reports the results of MI detection and localization, while the discussion and conclusion are offered in Section 4 and Section 5.

## Materials and Methods

### Datasets

We used the PhysioBank open access database (PTB Diagnosis ECG database) (Goldberger et al., 2000), which consists of standard 12-lead ECG data of 52 normal subjects (80 records) and 148 MI patients (368 records). The ECG signals were digitized at a sampling rate of 1,000 Hz. The MIs were divided into 10 types based on their localization: anterior (A), anterior lateral (AL), anterior septal (AS), inferior (I), inferior lateral (IL), inferior posterior (IP), inferior posterior lateral (IPL), lateral (L), posterior (P), and posterior lateral (PL). The database also includes labels on the ten types of MI and the localization.

### Data Preprocessing

**FIGURE 1:**
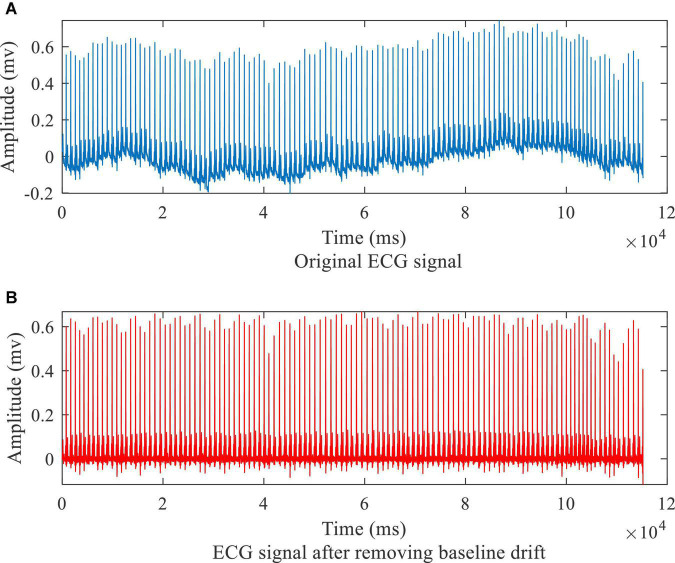
ECG signal before **(A)** and after **(B)** preprocessing.

**FIGURE 2:**
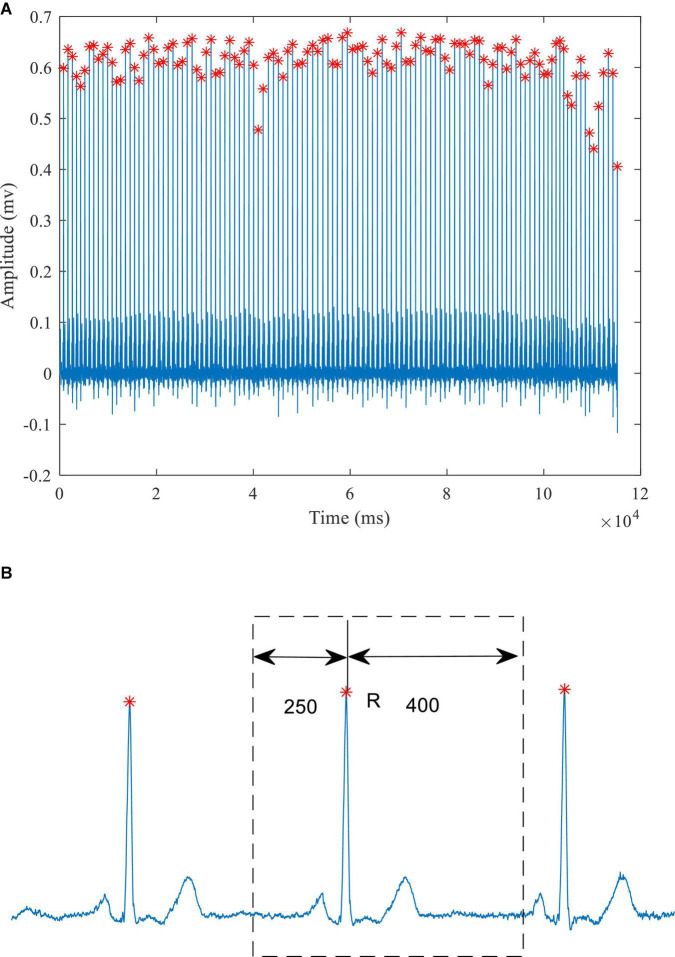
A typical ECG signal **(A)** Identification of the R-wave peaks (red crosses) **(B)** schematic diagram of heartbeat extraction.

The preprocessing of the ECG data consisted of two parts: removing baseline drift and segmenting the ECG signal to extract individual heart beats. As shown in Figure 1, a median filter over 333 sample points (333 ms) was used to remove baseline drift. The R waves were extracted, using the compute_hr function and the correct peaks function in the waveform-database (WFDB) package for Python. A typical example of the extracted R waves is shown in Figure 2A. When segmenting the ECG signals, each segment comprised a total of 651 sampling points, with 250 to the left of the R-peak (i.e., preceding it) and 400 to the right, as shown in Figure 2B (Baloglu et al., 2019). This window of 651 ms was sufficient to capture the QRS complex and the ST segment in all subjects. Table 1 lists the number of extracted heart beats from the 12-lead ECG.

**TABLE 1:** Number of 12-lead ECG beats analyzed for healthy subjects and each type of MI.

MI type	Number of beats
Healthy	112,800
Anterior	72,880
Anterior lateral	74,480
Anterior septal	137,680
Inferior	151,520
Inferior lateral	83,600
Inferior posterior	480
Inferior posterior lateral	27,840
Lateral	6,160
Posterior	6560
Posterior lateral	8,880
Total	682,880

### The Proposed ECGNet Model

**FIGURE 3:**
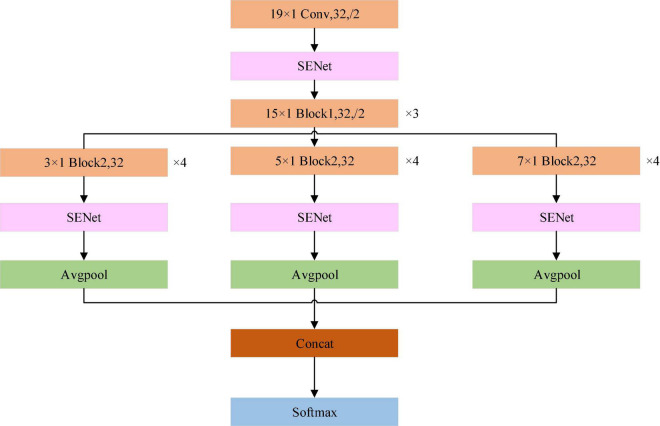
Schematic of the ECGNet model showing the individual modules. Avgpool is the average pooling layer. Concat is the operator to concatenate the features of different scales, SoftMax is the function which calculates the probability that the sample belongs to each category. The maximum probability determines that the sample is assigned to the specified class.

The structure of the proposed model is shown in Figure 3. The input to the model is a 651 × 12 array. Each channel of the 12-lead ECG is processed using the same convolution kernel. This approach can not only extract the common characteristics of the different leads, but also reduce the number of the model parameters. It is better to use a long convolution kernel model in ECG signal processing (Hannun et al., 2019). Therefore, at the start of the processing a relatively long kernel is adopted for feature extraction. As the network continues to extract features, the length of the convolution kernel can be reduced, which in turn reduces the computational burden. When the 12-lead ECG data is input to the model, the SENet model is applied to obtain the weighting of the signal from each lead. After the convolution operation is completed at each scale, the SENet model is again applied, this time to the eigenvector of each lead, and the weight information of each lead in the model after convolution is then updated.

**FIGURE 4:**
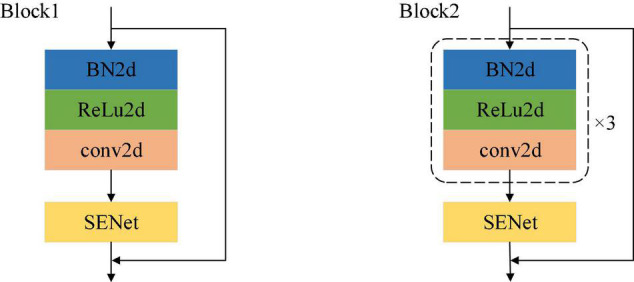
Schematic diagram of Block1 and Block2.

A schematic of the network modules of Block1 and Block2 is shown in Figure 4. As described by He et al. (2016) the improved residual module is adopted, and the order of the modules is batch normalization (BN), activation and convolution. In Figure 4, the only difference between the structures of Block1 and Block2 is that the processing steps are repeated three times in Block2.

This article adopts the channel attention mechanism proposed by the SENet network (Jie et al., 2017) for weight learning, and its structure is shown in Figure 4. Based on the previously designed multi-scale network, we have introduced the attention mechanism into the whole feature extraction process, so as to better learn local salient features, and have added the attention mechanism module to the ResNet module. The module is mainly realized by three operations: it can adaptively learn the weights of the various channel feature graphs. The first step is the squeeze operation. By means of global average pooling, the input feature map is compressed in the channel direction, and the two-dimensional feature map is transformed into a real number. Due to the global pooling, the obtained real number has a global receptive field. The second step is the exception operation, which corresponds to two full connection layers and one ReLU activation function layer. The weight is generated for each feature channel through the parameter W. The last step is the reweighting operation, which treats the weight of the exception output as the importance of each feature channel after feature selection, and then weights the input features channel-by-channel through multiplication, to complete the readjustment of the original features in the channel dimension.

The previously mentioned multi-scale network involves the sampling of different scales of the signal. Usually, we can observe different features at different scales to complete different tasks. Increasing the number of layers (deepening) of the convolutional neural network corresponds to the feature conversion process from low-level to high-level feature extraction. For an ECG signal, the shallow extraction of the network is generally low-level information such as waveform trends. As the network deepens, the extracted feature information may already be able to describe the entire ECG signal. However, each layer will lose some information in the process. To solve this problem, a solution of multi-scale feature fusion has appeared. The basic idea is to save the feature map of the previous layer and to add the feature map of the current layer, before the network convolution operation of the layer, so that some information from the previous layer can be retained and the loss of shallow information can be reduced.

The Class Activation Mapping (CAM) method has been proposed (Zhou et al., 2016). It is a visualization method that uses the activation of feature maps to understand neurons in the output layer. As the depth of the convolutional layer increases, the scattered detailed features are gradually transformed into overall contour features. The feature information extracted by the last convolutional layer is the most abundant, and CAM can use the information contained in that layer. The CAM calculates the weight matrix of the neural network by replacing the fully connected layer in the network with the global average pooling layer. It uses the weight matrix to weight the feature maps of the last layer, and to overlap the weighted feature maps into one matrix. The matrix is drawn and colored using a thermal imaging color scale and merged with the original input picture to intuitively display the area on which the model is based when making a judgment. However, because CAM requires a change in the structure of the model, it cannot be applied on a large scale to a well-trained depth model. Selvaraju et al. (2017) proposed Grad-CAM. For this approach, the idea of the CAM method is followed, but there is no need to change the original model and retrain. Instead, the gradient average is used to calculate the weight, and the deep learning model is visually displayed. Grad-CAM, as a generalized form of CAM, has a higher resolution as a thermal imaging color scale. The time series is used as the abscissa and the 12-lead ECG signal is used as the ordinate to convert one-dimensional signals into two-dimensional data.

The ECGNet model training incorporated a categorical cross entropy loss function and the Adam optimizer with learning rate of 0.0000008. The training process was stopped when there was no further reduction in the value of the loss function over 5 consecutive epochs using a batch size of 64. Moreover, for the MI localization or detection, the model that achieved the lowest error on the testing dataset was chosen. Hyper-parameters, including learning rate, batch size, and the number of epochs, seriously influence the overall performance detection and localization of MI. These were adjusted by trial and error. The highest validation accuracy and, as mentioned above, the lowest loss function were the criteria for selecting model parameters.

### Evaluation Metric

In order to evaluate the performance of the model on the testing data, we calculated the following commonly used evaluation criteria. These are accuracy (ACC), precision (PRE), sensitivity (SEN), specificity (SPE), and F1-Score. The TP, FP, TN, and FN are true positive, false positive, true negative and false negative, respectively. These are defined as follows:


(1) $$\text{ACC}=\frac{\mathrm{TP}+\mathrm{TN}}{\mathrm{TP}+\mathrm{FP}+\mathrm{TN}+\mathrm{FN}}$$

(2) $$\text{PRE}=\frac{\text{TP}}{\mathrm{TP}+\mathrm{FP}}$$

(3) $$\text{SPE}=\frac{\text{TN}}{\mathrm{TN}+\mathrm{FP}}$$

(4) $$\text{SEN}=\frac{\text{TP}}{\mathrm{TP}+\mathrm{FP}}$$

(5) $$\mathrm{F1}\,\text{-}\,\mathrm{Score}=\frac{2\times \text{SEN}\times \text{PRE}}{\mathrm{SEN}+\mathrm{PRE}}$$

## Results

**FIGURE 5:**
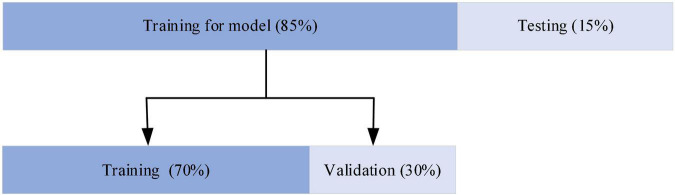
Distribution of the dataset among training, validation, and testing.

The network was developed in Python 3.6.9 using Keras and TensorFlow as the backend deep learning library. The workstation used for training the models consisted of two NVIDIA GeForce TRX 2080Ti, a 32 core CPU and 128 GB memory. This server ran a Linux system. As shown in Figure 5, for the evaluation of the model, 85% of the data were used for training and the remaining 15% for testing. The model training data are then partitioned for training and validation in the ratio of 7:3. The selection of training and test 12-lead ECG frames for the ECGNet approach was performed using five-fold cross validation techniques (Sharma et al., 2018).

### Myocardial Infarction Detection

For the detection of MI, each ECG signal was classified as one of only two types, healthy or MI. Using five-fold cross validation, the evaluation indexes of the two types of heartbeat were calculated, according to eqs. (1–5). The results of the MI detection are listed in Table 2. The average accuracy, sensitivity, specificity, precision, and F1-score of the detection of HC based on the proposed method were 99.98, 99.87, 99.99, 99.99, and 99.93%, respectively. The average accuracy, sensitivity, specificity, precision, and F1-score of the detection of MI based on the proposed method were 99.98, 99.99, 99.87, 99.98, and 99.98%, respectively.

**TABLE 2:** Results of five-fold cross validation for MI detection (HC denotes healthy controls).

Folds	Category	ACC (%)	SEN (%)	SPE (%)	PRE (%)	F1-score
Fold 1	HC	99.94	99.72	99.99	99.93	99.82
	MI	99.94	99.99	99.72	99.94	99.96
	Average	99.94	99.86	99.86	99.94	99.89
Fold 2	HC	99.98	99.86	100.00	100.00	99.93
	MI	99.98	100.00	99.86	99.97	99.99
	Average	99.98	99.93	99.93	99.99	99.96
Fold 3	HC	99.99	99.93	100.00	100.00	99.96
	MI	99.99	100.00	99.93	99.99	99.99
	Average	99.99	99.97	99.97	100.00	99.98
Fold 4	HC	99.99	99.93	100.00	100.00	99.96
	MI	99.99	100.00	99.93	99.99	99.99
	Average	99.99	99.97	99.97	100.00	99.98
Fold 5	HC	99.99	99.93	100.00	100.00	99.96
	MI	99.99	100.00	99.93	99.99	99.99
	Average	99.99	99.97	99.97	100.00	99.98

As shown in Table 3, there are 8,536 samples in the test data set, among which 7,123 samples of MI were correctly identified, with an average sensitivity of nearly 100%, and a low rate of missed detection. Among the 1,410 healthy samples, 1408 were correctly identified and 2 were wrongly classified as MI, demonstrating the low false detection rate. The results as shown by the confusion matrix confirm the excellent performance.

**TABLE 3:** Confusion matrix for MI detection.

Original/Predicted	MI	HC
**MI**	7,125	2
**HC**	1	1,408

### Myocardial Infarction Localization

The classification results of the localization of MI using five-fold cross validation are listed in Table 4. For inferior posterior and lateral infarctions, the average sensitivity, specificity, precision, F1-score were 100%; the classification performance of other categories was also excellent. Anterior MIs, were detected with the lowest sensitivity (99.47%), the lowest specificity (99.94%), the lowest precision (99.45%) and the lowest F1-score (99.46%). These results strongly support the effectiveness of the proposed model. During the training process, the accuracy of the loss function and training set tended to be stable after 25 epochs. Finally, the accuracy of the training set was 100%, the accuracy of the verification set was 99.86%, the loss of the verification set was 0.02413, and the accuracy of the final test was 99.89%. In order to show the performance of the model in more detail, the confusion matrix from the test set is given in Table 5. Among the 8536 samples in the test set, 8,527 were correctly classified, and 1410 normal ECGs were correctly classified as non-MI samples, so the false detection rate too, was very low. The missed detection rate was, similarly, very low, with only one MI sample classified as healthy. In only 8 of the 7,126 MI samples, was the localization wrongly identified, although in each case the presence of an MI was correctly detected. The localization error rate was 0.11%, confirming the excellent performance in detecting the position of the MIs. The accuracy, sensitivity and specificity were 99.79, 99.88, and 99.98%, respectively.

**TABLE 4:** Classification performance of MI localization (five-fold cross validation).

Category		SEN (%)	SPE (%)	PRE (%)	F1-score (%)	Number of beats
Healthy	Fold 1	99.93	99.96	99.93	99.93	1,410
	Fold 2	100	99.96	100	100	1,410
	Fold 3	99.92	99.96	99.93	99.93	1,410
	Fold 4	99.93	99.97	99.86	99.89	1,410
	Fold 5	99.93	100	100	99.96	1,410
	**Average**	**99.94**	**99.97**	**99.94**	**99.94**	**1,410**
Anterior	Fold 1	99.45	99.93	99.45	99.45	911
	Fold 2	99.34	99.93	99.34	99.34	911
	Fold 3	99.56	99.96	99.67	99.62	911
	Fold 4	99.45	99.93	99.45	99.45	911
	Fold 5	99.56	99.93	99.34	99.45	911
	**Average**	**99.47**	**99.94**	**99.45**	**99.46**	**911**
Anterior lateral	Fold 1	99.79	99.97	99.79	99.79	931
	Fold 2	99.88	99.97	100.00	99.84	931
	Fold 3	99.79	99.96	99.68	99.73	931
	Fold 4	99.68	100.00	100.00	99.84	931
	Fold 5	99.79	99.90	100.00	99.89	931
	**Average**	**99.79**	**99.96**	**99.89**	**99.82**	**931**
Anterior septal	Fold 1	99.71	99.90	99.59	99.65	1,721
	Fold 2	99.95	99.90	99.54	99.59	1,721
	Fold 3	99.97	99.93	99.71	99.71	1,721
	Fold 4	99.88	99.88	99.54	99.71	1,721
	Fold 5	99.59	99.93	99.59	99.59	1,721
	**Average**	**99.82**	**99.91**	**99.59**	**99.65**	**1,721**
Inferior	Fold 1	99.74	99.98	99.95	99.84	1,894
	Fold 2	99.96	99.98	99.84	99.76	1,894
	Fold 3	99.89	99.95	99.84	99.87	1,894
	Fold 4	99.63	99.97	99.89	99.76	1,894
	Fold 5	99.63	99.97	99.79	99.71	1,894
	**Average**	**99.77**	**99.97**	**99.86**	**99.79**	**1,894**
Inferior lateral	Fold 1	99.90	100.00	100.00	99.95	1,045
	Fold 2	100.00	100.00	99.71	99.86	1,045
	Fold 3	99.81	99.99	99.90	99.86	1,045
	Fold 4	99.63	99.97	99.90	99.95	1,045
	Fold 5	99.90	99.95	99.62	99.76	1,045
	**Average**	**99.85**	**99.98**	**99.83**	**99.88**	**1,045**
Inferior posterior	Fold 1	100.00	100.00	100.00	100.00	6
	Fold 2	99.99	100.00	100.00	100.00	6
	Fold 3	100.00	100.00	100.00	100.00	6
	Fold 4	100.00	99.99	100.00	100.00	6
	Fold 5	100.00	100.00	100.00	100.00	6
	**Average**	**100.00**	**100.00**	**100.00**	**100.00**	**6**
Inferior posterior lateral	Fold 1	100.00	99.98	99.43	99.71	348
	Fold 2	100.00	99.98	99.71	99.71	348
	Fold 3	100.00	100.00	100.00	100.00	348
	Fold 4	100.00	100.00	100.00	100.00	348
	Fold 5	99.71	99.98	99.43	99.57	348
	**Average**	**99.94**	**99.99**	**99.71**	**99.80**	**348**
Lateral	Fold 1	100.00	100.00	100.00	100.00	77
	Fold 2	100.00	100.00	100.00	100.00	77
	Fold 3	100.00	100.00	100.00	100.00	77
	Fold 4	100.00	100.00	100.00	100.00	77
	Fold 5	100.00	100.00	100.00	100.00	77
	**Average**	**100.00**	**100.00**	**100.00**	**100.00**	**77**
Posterior	Fold 1	100.00	99.99	98.80	99.39	82
	Fold 2	100.00	99.99	98.80	100.00	82
	Fold 3	100.00	100.00	100.00	100.00	82
	Fold 4	100.00	100.00	100.00	100.00	82
	Fold 5	100.00	100.00	100.00	100.00	82
	**Average**	**100.00**	**100.00**	**99.52**	**99.88**	**82**
Posterior lateral	Fold 1	100.00	100.00	100.00	100.00	111
	Fold 2	99.99	100.00	99.11	99.55	111
	Fold 3	100.00	100.00	100.00	100.00	111
	Fold 4	100.00	100.00	100.00	100.00	111
	Fold 5	100.00	100.00	100.00	100.00	111
	**Average**	**100.00**	**100.00**	**99.82**	**99.91**	**111**

**TABLE 5:** Confusion matrix for MI localization.

Original/Predicted	H	A	AL	AS	I	IL	IP	IPL	L	P	PL
**H**	1410	0	0	0	0	0	0	0	0	0	0
**A**	0	910	0	0	0	1	0	0	0	0	0
**AL**	0	0	929	2	0	0	0	0	0	0	0
**AS**	1	0	0	1719	1	0	0	0	0	0	0
**I**	0	2	0	2	1890	0	0	0	0	0	0
**IL**	0	0	0	0	0	1045	0	0	0	0	0
**IP**	0	0	0	0	0	0	6	0	0	0	0
**IPL**	0	0	0	0	0	0	0	348	0	0	0
**L**	0	0	0	0	0	0	0	0	77	0	0
**P**	0	0	0	0	0	0	0	0	0	82	0
**PL**	0	0	0	0	0	0	0	0	0	0	111

The signals from different leads are correlated, but the signal of each lead is one-dimensional, so a two-dimensional convolution is not applicable. Inspired by the concept of multi-sensor data fusion, we treated the data from all the leads as a two-dimensional by combining the one-dimensional signals from all 12-leads. Thus, each beat of the 12-lead signal was represented by a 12 × 651 array. To extract abundant feature information effectively by using a two-dimensional convolution, three different convolution kernels were used, these being 3 × 1, 5 × 1 and 7 × 1. Taking the 7 × 1 kernel as an example, seven consecutive sampling points from the same lead were extracted in one convolution.

**FIGURE 6:**
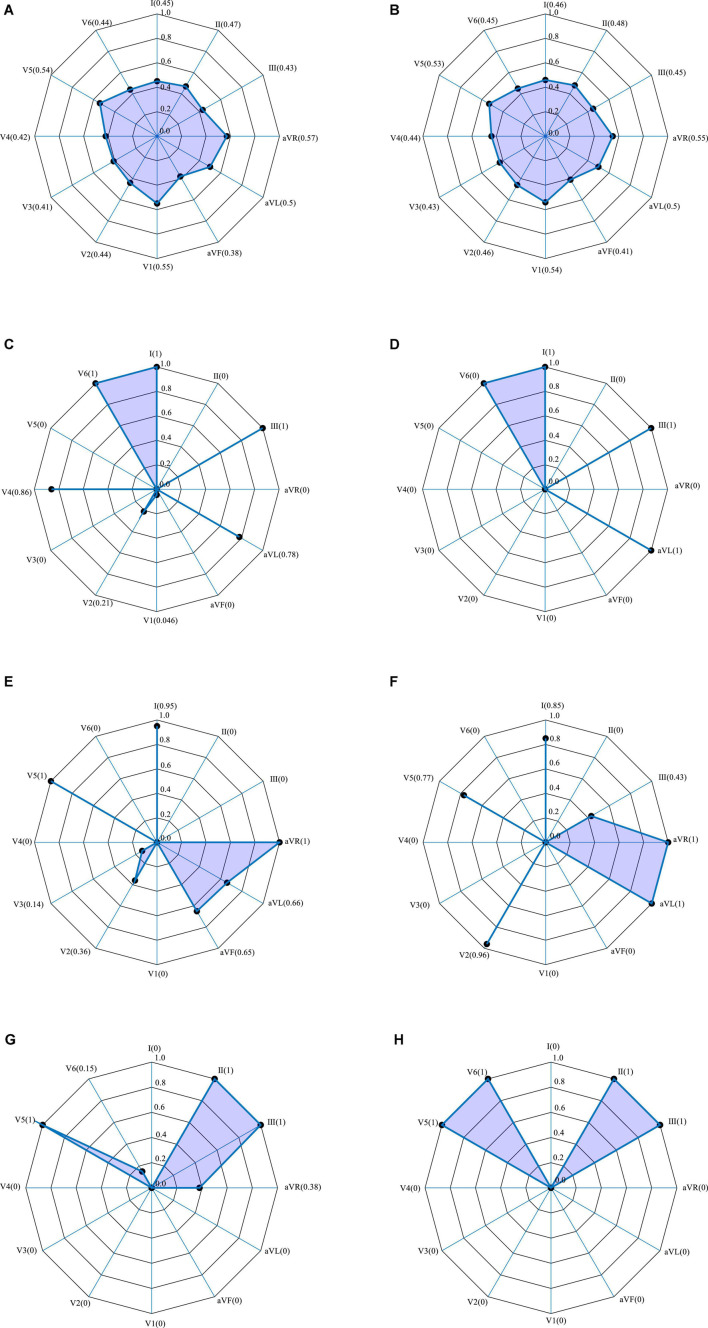
12-lead ECG weightings obtained by SENet. Polar plots from two representative infarctions. **(A–G)** Anterior MI: **(A)** without convolution; **(C)** with 3 × 1 convolution; **(E)** with 5 × 1 convolution; **(G)** with 7 × 1 convolution. **(B–H)** Inferior MI: **(B)** without convolution; **(D)** with 3 × 1 convolution; **(F)** with 7 × 1 convolution; **(H)** with 7 × 1 convolution.

According to the association between the localization of the MI and the pattern of signals from the 12 leads ECG, the SENet model automatically calculates the weighting of each lead at the first stage of the model and again after three different convolution orders. Figure 6 shows the results of this procedure using representative anterior and inferior MIs as examples. Initially, with an un-convolved input, there is little variation in the weighting of each lead, with values between 0.4 and 0.6, as shown in Figures 6A,B. Following convolution, the lead weightings fall into two patterns according to the convolution order. As shown in Figure 6C, for the of 3 × 1 convolution kernel, the weights of leads V6, I, III, and AVL are larger than the average, and the weights of the other leads are smaller. This implies that the model “thinks” the features acquired from these four leads are more important. Figure 6E shows that, for the 5 × 1 convolution kernel, the weights of leads I, V5, and AVR are generally larger, suggesting that the characteristics of these leads are more important. In addition, the common localizations of different infarct types will have an effect. For example, the weights of AVL and V2 are, respectively, 1 and 0.97 for the inferior infarction in Figure 6F. The weighting information obtained by the three convolution scales is quite different, which confirms the notion that the multi-scale model extracts different levels of feature information from the data, and that the feature set is more abundant after feature fusion.

**FIGURE 7:**
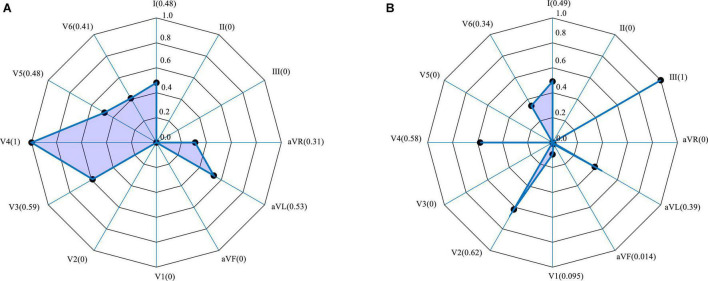
Lead weighting distribution obtained by Grad-CAM for two representative MIs. **(A)** Anterior; **(B)** inferior.

Figure 7 shows the weighting of each lead derived from the Grad-CAM model trained using 406313 samples (i.e., 70% of the 580448 samples used for training). A typical anterior MI is shown in Figure 7A, and a posterior case, in Figure 7B. For the anterior case, the relative lead weights are ordered from high to low as follows: V4, V3, AVL, V5, I, V6, AVR, II, III, V1, V2, and AVF, where the weights of the lowest 4 (II, III, V1, V2, and AVF) are 0. For the posterior MI, the order is: III, V2, V4, I, AVL, V6, V1, AVF, II, AVR, V3, and V5, where the weights of the lowest 3 (AVR, V3, and V5) are 0. In the pathology of MI, leads V3 and V4 are associated with the anterior wall of the heart, while leads II, III and AVF are associated with the posterior wall. Thus, V3 and V4 contribute the most to the detection of anterior MIs, while lead III contributes the most to the detection of posterior MIs (Chang et al., 2012). It can be concluded that lead weighting analysis can provide diagnostic information about the localization of the MI. However, due to the limited sample size in this study and variability between patients, the results cannot fully explain the pathological significance of the features extracted by the model.

## Discussion

### Myocardial Infarction Detection

Table 6 shows that several current approaches, some of which are briefly discussed below, also perform well in the detection of MI. Dohare et al. (2018) extracted the peak amplitude, area, average and standard deviation of the P wave, the QRS band, QT interval and ST segment of 12-lead ECG signals to act as the feature set for MI detection. After screening, the features were passed to the support vector machine for training, achieving an MI detection accuracy of 96.66%. The performance of the algorithm was closely related to the accuracy of prior segmentation of the ST segment, T wave, Q wave, and P wave. However, the characteristic ECG changes in patients with MI will vary with the time since the MI occurred. This increases the difficulty of prior segmentation of the ECG signal, and greatly affects the ability to accurately detect MIs.

**TABLE 6:** Comparison of MI detection results from this study with other recent reports.

Study	Lead	ACC (%)	SEN (%)	SPE (%)
Dohare et al. (2018)	12 leads	96.66	96.66	96.66
Kumar et al. (2017)	II lead	96.84	95.80	97.60
Sharma et al. (2018)	Signal lead	With noise: 99.62 Without noise: 99.74	With noise: 99.76 Without noise: 99.84	With noise: 92.83 Without noise: 94.19
Acharya et al. (2017)	II lead	With noise: 93.53 Without noise: 95.22	With noise: 93.71 Without noise: 95.49	With noise: 92.83 Without noise: 94.19
Strodthoff and Strodthoff (2019)	12 leads	–	93.30	89.70
Liu et al. (2018)	12 leads	99.95	–	–
Lui and Chow (2018)	I lead	–	92.40	97.70
Tripathy et al. (2019a)	12 leads	99.74	99.87	99.60
**This study**	**12 leads**	**99.98**	**99.94**	**99.94**

*ACC, accuracy; SEN, sensitivity; SPE, specificity.*

Kumar et al. (2017) used the flexible wavelet transform to map the ECG signal of lead II to multidimensional space, then extracted the sample entropy associated with each frequency band of the signal as the feature of interest. Sharma et al. (2018) used the improved wavelet transform to decompose the ECG signal and extract the entropy of each sub-band to form the feature set. Combined with the KNN method, they achieved a high level of precision in recognizing MI from noise-free as well as noisy signals (Sharma et al., 2018). Although this kind of approach using feature space to extract useful features has achieved satisfactory results, problems remain, such as the need for manual feature selection, and lack of applicability to the ECG signals associated with a wide range of MI types.

Deep learning has solved the problem of the lack of generalizability inherent in manual feature extraction, traditionally used in MI detection. Lui and Chow (2018) achieved excellent performance with a combination of a convolutional neural network and a recurrent neural network, giving a sensitivity and specificity of 92.40 and 97.70%, respectively. Tripathy et al. (2019a) performed a Fourier-Bessel series expansion-based empirical wavelet transform on 12-lead ECG signals to extract useful features for detection of MIs, achieving an accuracy, sensitivity, and specificity of 99.74, 99.87, and 99.94%, respectively.

The multi-scale ResNet approach to detect MI proposed in this study, differs from the methods described in Table 6. The proposed method does not require prior segmentation, manual feature extraction or manual selection of features. It automatically extracts relevant feature information, and automatically learns which are the important features for detecting MI, through the SENet model which, accordingly, adjusts the weighting value of each lead to obtain better classification accuracy. As shown in Table 6, the experimental results show that the accuracy, sensitivity, and specificity of our approach are higher than those achieved by others, thus verifying the effectiveness of the method described here. It is notable that the residual network based on a multi-scale approach has enriched the comprehensive features of the ECG signal and the channel attention mechanism has enhanced the important features automatically.

### Myocardial Infarction Localization

The position of an MI can be found by analyzing its effect on different ECG leads, especially those closest to the infarction. A previous study analyzing the relationship between infarction position and characteristic lead patterns, obtained good results (Fu et al., 2020). However, it did not exploit this further to gain information about the localization of the lesion. In recent years, the attention mechanism has attracted extensive interest in clinical diagnosis and the deep fusion attention mechanism model has been used to extract complex features from physiological signals for disease classification and diagnosis (Yuan and Jia, 2019). In the diagnosis of MI, the attention mechanism based on a beat-by-beat analysis has been introduced to automatically calculate the relative weighting of unlabeled beats (Zhang et al., 2019). The attention mechanism has strong explanatory power. Not only can it calculate the contribution of different extracted features to the results, but it can also calculate the weighting of multiple lead signals.

Table 7 briefly summarizes some recent studies on the localization of MI from ECG signals. Some methods are based on traditional manual feature extraction algorithms, which mainly yield features in the time and transform domains, while others extract features automatically, using deep learning approaches. As listed in Table 7, recent studies have achieved good results in the localization of MI. Arif et al. (2012) manually extracted morphological features from 12-lead ECG signals, such as the T wave, Q wave and ST segment amplitude and combined these with KNN classifier training, obtaining an MI localization accuracy of 99.8%. Acharya et al. (2016) used the discrete wavelet transform to map the ECG signal to a 9-layer scale space, where each layer extracted 12 kinds of non-linear entropy as features. These were sent to a KNN classifier for localizing MIs further. The accuracy, sensitivity and specificity were 98.74, 99.55, and 99.16%, respectively. Liu et al. (2018) used the wavelet transform to process individual beats of the ECG signal to construct a wavelet tensor. This was combined with a decision tree classifier to realize the localization of the MI, obtaining an accuracy, of 99.81%. Barmpoutis et al. (2019) also used the wavelet transform to construct tensor data, but in this case mapped them to Euclidean space and Glassman space for feature extraction and then fused these two different feature representations and the complementary information produced by each, into a common Hilbert space for classification. This resulted in the localization of MIs with an accuracy of 100%, although they identified only 5 MI localizations and did not report sensitivity or specificity figures. In the clinical environment, the presence of an MI will lead to complex waveform changes of multi-lead ECG signals, and different patients will have different ECG waveforms. In spite of their impressive results, these studies do not lend themselves well to widespread clinical use because, due to differences between patients, even those with the same pathology, they require manual intervention and in many cases are not able to provide detailed localization data. In comparison with traditional machine learning, deep learning is more effective in recognizing features and is better able to identify features specific to each type of MI, even in the face of variability between patients. The residual network based on multiple scale and the channel attention mechanism in our proposed approach can extract more information and more effectively identify the important features.

**TABLE 7:** Comparison of MI localization results from recent reports with those from this study.

Study	Lead	Number of MI localizations studied	ACC (%)	SEN (%)	SPE (%)
Sun et al. (2012)	12 leads	5	–	85.00	–
Arif et al. (2012)	12 leads	11	98.8	–	–
Acharya et al. (2016)	12 leads	11	98.74	99.55	99.16
Padhy and Dandapat (2017)	12 leads	6	98.1	–	–
Le et al. (2013)	3 leads	6	–	88.00	92.00
Sharma et al. (2015)	12 leads	6	99.58	–	–
Barmpoutis et al. (2019)	12 leads	5	100	–	–
Liu et al. (2018)	12 leads	6	99.81	–	–
Baloglu et al. (2019)	12 leads	11	99.78	–	–
Tripathy et al. (2019b)	12 leads	7	99.84	–	–
**This study**	**12 leads**	**11**	**99.79**	**99.88**	**99.98**

*ACC, accuracy; SEN, sensitivity; SPE, specificity. Note, up to 10 localizations studied, plus healthy controls.*

Furthermore, MI localization algorithms based on deep learning can, in principle, solve the problem of manual feature extraction used in traditional approaches, because feature information can be obtained automatically. Zhang et al. (2019) used the combination of sparse encoder and bagged decision tree to automatically extract the features of single lead ECG signal and obtained accuracy, sensitivity, and specificity of MI localization of 98.88, 99.95, and 99.87%, respectively. Baloglu et al. (2019) used a deep convolutional neural network model to automatically identify 12-lead ECG signals and obtained a positional accuracy of 99.78%. However, although these methods have achieved good performance in MI localization, most of them assume that each lead contributes equally to the results and do not therefore exploit the fact that each lead’s signal contains unique information about the position of the lesion causing the MI.

Unlike the other localization algorithms described in Table 7, the multi-scale ResNet approach based on multi-lead ECG signals proposed in this paper, does not need to extract the wave features prior to locating the MI, and fully considers the similarities and difference amongst different leads to extract their features. Having identified these common features, the SENet model then automatically calculates the weighting for each lead (dependent on the localization of the MI) after feature extraction, using various convolution kernels. The average accuracy, sensitivity and specificity of the proposed method are 99.79, 99.88, and 99.98%, respectively, and it can reliably identify 10 types of MI as well as those patients without an infarction. At the same time, the ECGNet extracts rich feature information at different levels, reduces the number of parameters, and embodies an efficient and concise positioning model. This is enhanced by assigning and considering the weighting of each lead. The results show that the approach is ideal for inferring the localization of an MI from a 12-lead ECG, although further verification with a wide range of clinical data is necessary.

The detection and localization of MI based on deep learning applied to ECG signals proposed in this work has achieved good results, although there are still some limitations. At present, most studies of this type, including this one, are based on public databases. Future work to address this limitation should include an expanded dataset containing more clinical data to improve the generalizability of the model. A second limitation is that the algorithm used by the SENet model relies only on the differences between leads in signal amplitude to identify the various types of myocardial infarction. In future work, we will apply the analysis to different segments of the cardiac cycle, rather than the entire heartbeat.

## Conclusion

Computer aided diagnosis of myocardial infarction remains a challenging topic. During the training, the weighting information for the 12-lead is calculated using the SENet model, Grad-CAM algorithm, and combined with clinical and pathological experience of myocardial infarctions. The results of the model are based on the lead level, rather than analysis of each beat, as is the case in other studies. The process automatically extracts relevant feature information, and through the SENet model, automatically learns important features for detecting MI, and by adjusting the weighting of nearby leads achieves an accurate and robust determination of the lesion’s localization. Ten types of myocardial infarction are diagnosed from the 12-lead ECG signals. We conclude that the multi-scale deep learning model based on a residual network and attention mechanism proposed here, is an effective method to detect and locate MI and after further testing and validation on a larger number of cases, will provide a significant addition to the field of automatic ECG-based cardiovascular diagnosis.

## Data Availability Statement

Publicly available datasets were analyzed in this study. This data can be found here: https://www.physionet.org/content/ptbdb/1.0.0/.

## Ethics Statement

Ethical review and approval was not required for the study on human participants in accordance with the Local Legislation and Institutional Requirements. Written informed consent for participation was not required for this study in accordance with the National Legislation and the Institutional Requirements.

## Author Contributions

YC, WL, and LX proposed the scientific problems. YC, WL, SZ, BZ, and HC processed the data and conducted statistical analysis. NG and HH provided guidance on medical science issues. WL, SG, and LX contributed to the revision and final version of the manuscript. All authors reviewed the manuscript and read and approved this submission.

## Conflict of Interest

The authors declare that the research was conducted in the absence of any commercial or financial relationships that could be construed as a potential conflict of interest.

## Publisher’s Note

All claims expressed in this article are solely those of the authors and do not necessarily represent those of their affiliated organizations, or those of the publisher, the editors and the reviewers. Any product that may be evaluated in this article, or claim that may be made by its manufacturer, is not guaranteed or endorsed by the publisher.

## Abbreviations

A: anterior
ACC: accuracy
AL: anterior lateral
AS: anterior septal
BN: batch normalization
CNN: convolutional neural networks
ECG: electrocardiogram
Grad-CAM: gradient class activation mapping
I: inferior
IL: inferior lateral
IP: inferior posterior
IPL: inferior posterior lateral
KNN: K-nearest neighbors algorithm
L: lateral
LSTM: long short-term memory
MI: myocardial infarction
P: posterior
PL: posterior lateral
PRE: precision
RNN: recurrent neural networks
SPE: specificity
SVM: support vector machine.
